# Effects of information and communication technology on total-factor carbon emission efficiency and the health co-benefits

**DOI:** 10.3389/fpubh.2023.1301627

**Published:** 2024-01-05

**Authors:** Jingying Linghu, Michal Wojewodzki, Tsun Se Cheong

**Affiliations:** ^1^Business School, Beijing Normal University, Beijing, China; ^2^College of Business, University of Doha for Science and Technology, Doha, Qatar; ^3^School of Business, The Hang Seng University of Hong Kong, Hong Kong, Hong Kong SAR, China

**Keywords:** information and communication technology development, total-factor carbon emission efficiency, health, dynamic threshold model, moderating effect

## Abstract

Information and communication technology (ICT) has great potential to propel economic development toward a low-carbon direction. This study aims to investigate the effect of ICT development on total-factor carbon emission efficiency (TFCEE), as well as its public health co-benefits. We use dynamic (threshold) models and a panel of 30 Chinese provinces from 2008 to 2019. The results suggest that ICT significantly and positively impacts the TFCEE. Specifically, for every 10 per cent increase in the internet development index, the TFCEE increases by 0.11 per cent. Moreover, we find that ICT development indirectly improves the TFCEE by promoting green innovation and energy structure optimization. Furthermore, when green innovation (energy structure represented by the share of coal) switches from below to above (above to below) its threshold value, the promotion effect of ICT development on the TFCEE increases. Additionally, the results show that improving the TFCEE can lead to co-benefits in strengthening China's public health. This study delivers novel insights on promoting the TFCEE through the ICT channel and highlights its positive health-related externalities. Furthermore, we offer policy recommendations to Chinese decision-makers, which can apply to other emerging economies battling similar issues.

## 1 Introduction

While China's economy has grown significantly during the past four decades, its carbon emissions have increased dramatically. Consequently, China is the world's leading greenhouse gas emitter (1). Ample empirical evidence indicates that carbon emissions are among the significant factors threatening public health (2–4). Thus, low-carbon transition has massive potential to bring public health co-benefits by directly reducing carbon dioxide inhalation, mitigating climate change, and reducing air pollutants that share emission sources with CO_2_ (2, 5–7). Against the above backdrop, Chinese policymakers pledged to peak total CO_2_ emissions by 2030 and to achieve carbon neutrality by 2060 (8) to maintain a balance between sustainability and carbon emissions. It is estimated that under the conditions of achieving the 1.5 C temperature goal and reaching the peak earlier than 2030, Chinese policymakers could prevent 118,000 (614,000) deaths by 2030 (2050) (9).

Notwithstanding, as an emerging economy, China needs to promote prolonged economic expansion. Thus, in the face of conflicting goals of carbon emissions reduction and continued economic growth, the key to resolving this dilemma is to improve the total-factor carbon emission efficiency (hereafter TFCEE) (10). TFCEE takes the GDP (carbon emissions) as the desired (undesired) output while considering the factor inputs of capital, labor and energy. As such, the TFCEE can effectively measure whether and to what extent regional development models meet the requirements of low-carbon transformation (11). Given the above backdrop, how to effectively improve the TFCEE has become a topical research question in recent environmental studies (10, 12).

**Figure 1:**
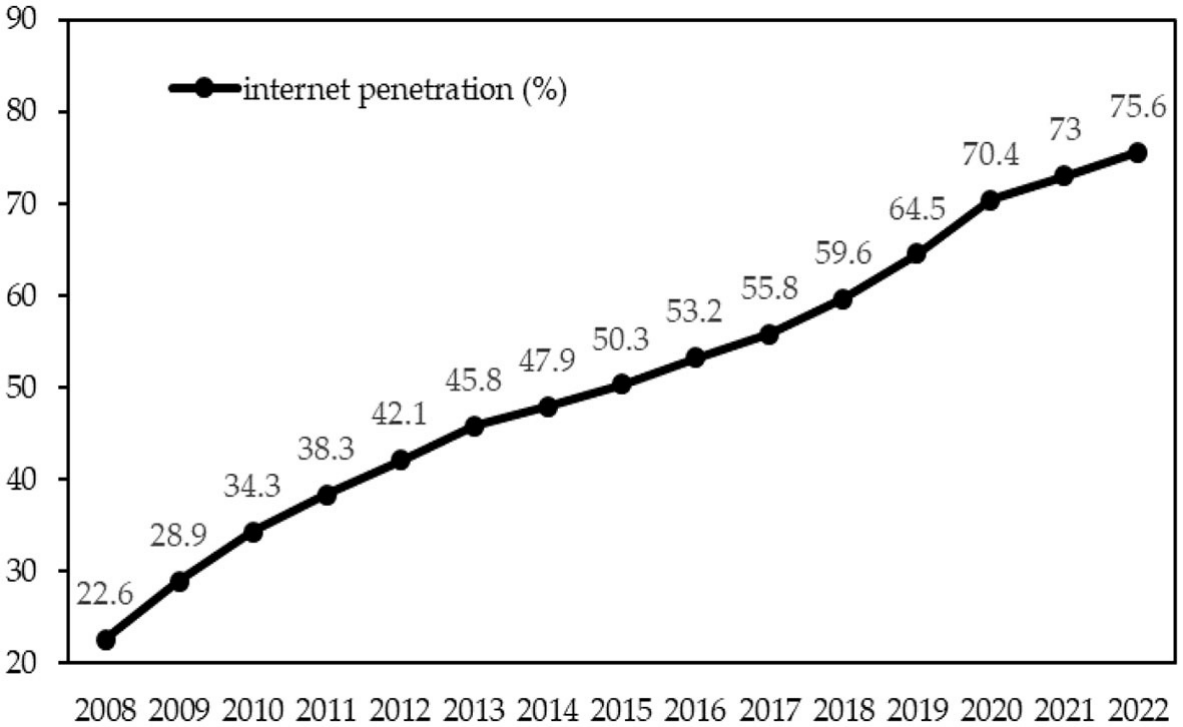
China's internet penetration growth in percentage terms from 2008 to 2022. Source: CNNIC, 2008-2022.

Since the 21^st^ century, the integration of information and communication technology (hereafter ICT) with various sectors of the economy has shown an unstoppable trend. Countries worldwide endeavor to develop and harness ICT, with internet technology being an essential part of it. China's public internet access started in 1994, while in August 2013, the State Council issued the Broadband China policy vigorously promoting ICT infrastructure construction. The recent 50^th^ Statistical Report released by the China Internet Network Information Center (13) shows that, between 2008 and 2022, internet penetration in China has more than tripled from 22.6 to 75.6 per cent (see Figure 1).

Many studies investigate the effect of ICT or internet development on variables related to energy or carbon emissions, but the results remain inconclusive. Some scholars argue that ICT-related factors reduce carbon intensity (14). Others find that internet development increases enterprise energy efficiency (15) and improves the TFCEE (11, 16). On the contrary (17, 18), document that demand for ICT products increases carbon emissions and energy intensity. Overall, the existing literature has four important shortcomings. First, current studies ignore the suspected path dependence of TFCEE and use static regression models, which may yield estimation bias (11, 16). Second, the mediation and threshold effects between ICT development and TFCEE have not received adequate attention. Third, while limited studies show a positive (negative) association between ICT (carbon emissions) and public health in China (2, 9, 19), the health co-benefits from improving TFCEE remain unexplored.

Given the pivotal role of ICT in propelling China's economic development toward a low-carbon direction and the above-listed gaps in empirical research, our study has four main objectives. First, to investigate the relationship between ICT and TFCEE using a dynamic model. Second, to unveil how ICT impacts TFCEE through the mediation effect of green innovation and energy structure optimization and the threshold effect of ICT at different values of the two mediator variables. Finally, the study directly examines whether and how TFCEE improvement affects public health across 30 Chinese provinces.

Accordingly, the contributions of this study are as follows. We are the first to employ the dynamic model to identify the role of ICT in promoting TFCEE. Specifically, we use the two-step system generalized method of moments (hereafter 2SGMM), which accounts for the suspected path dependence (autoregressive process) of the TFCEE variable and mitigates the endogeneity problem. Moreover, we document nascent empirical evidence of the significantly beneficial effect of ICT on the TFCEE via its positive influence on green innovation and energy structure optimization. Additionally, we are the first to quantify the significantly different impact of ICT on the TFCEE concerning threshold levels of green innovation and energy structure. Finally, we deliver a novel contribution by documenting positive public health-related externalities associated with policies that improve the TFCEE through ICT development.

The remainder of this article is as follows: Section 2 presents a literature review. Section 3 develops the hypotheses, while Section 4 offers data and research methods. In Section 5, we outline and discuss the empirical results. We conclude the study and provide policy suggestions in Section 6.

## 2 Literature review

### 2.1 TFCEE measurements

One can measure carbon emission efficiency by single-factor indicators, which usually reflect the interaction between carbon emissions and GDP. For example, CO_2_ emissions intensity is the ratio of carbon emissions to GDP (1). However, single-factor indicators do not consider all the key inputs in the production process, as well as the substitution effect among inputs. Thus, single-factor indicators are not capable of fully measuring CO_2_ emission efficiency (10). Because of this limitation, some studies add capital and labor inputs into the analysis of carbon emission efficiency and propose the TFCEE as an alternative superior indicator (20).

Using TFCEE, we can estimate more accurately whether the development pattern meets the sustainable requirement of economic growth and carbon abatement. Data envelopment analysis methods are widely used to calculate the TFCEE (10). For instance, the directional distance function (hereafter DDF) captures the production process, including the undesired output of carbon emissions (21). However, the original DDF model is limited to increasing (reducing) desired (undesired) outputs (and/or inputs) with the same ratio, thereby leading to an overestimation of efficiency when non-zero slacks exist (22). Therefore, scholars propose the non-radial directional distance function (hereafter NDDF) based on slack variables to solve such an acute problem (23).

### 2.2 ICT development and TFCEE nexus

Scholars examining the multifaceted effects of ICT/internet advancements on energy usage or carbon emissions deliver conflicting evidence. On the one hand, some researchers find that ICT/internet development reduces carbon emissions (24), CO_2_ per capita (25), carbon intensity (14), and energy consumption (26) while improving energy efficiency (27). On the other hand, others argue that the ICT/internet threatens the sustainability of resources and the environment by increasing CO_2_ emissions (17) and energy intensity (18). Moreover (28), point out that the impact of ICT on carbon emissions per capita is uncertain by documenting a positive (negative) relationship in the short (long) term. It is worth noting that the studies mentioned above use static models. However, the present value of the TFCEE is significantly influenced by its earlier values, which gives rise to a robust cumulative circularity (12, 29).

Two recent studies exploring the ICT and TFCEE nexus in China use static models and reveal a positive relationship (11, 16). Notwithstanding, some research suggests that there may be a threshold effect concerning the relationship between ICT progress and the environment or energy (30, 31). However, to our knowledge, no studies have investigated the threshold effects in the context of ICT effects on the TFCEE.^1^ Instead, researchers primarily use simple multivariate linear models, neglecting the jumping character or structural breaks in the relationship between ICT development and the TFCEE caused by changes in other economic variables (11, 16).

Moreover, the relationship between the environment and health has long been one of the research hotspots (9, 32, 33). Studies examining China (1), the United States (2), and OECD countries (3) document the adverse effects of carbon dioxide on residents' health. However, there is a considerable lack of research on the impact of TFCEE on health. Additionally, pregnant women are an environmentally sensitive group (34), and health literature has found a strong correlation between air pollution and maternal complications and mortality risk factors (35). However, the maternal group is often overlooked in environmentally related studies.

### 2.3 Research gaps

Summing up, the following deficiencies are found in the relevant empirical literature: (1) Existing research has not considered that the TFCEE is usually path-dependent (autoregressive process) and has used static models for estimation. (2) Few studies focus on the mediation and threshold effects between ICT development and the TFCEE. (3) Health benefits of the TFCEE improvement remain under-explored. Our study attempts to fill the above research gaps.

## 3 Hypotheses development

### 3.1 The direct effect of ICT development on the TFCEE

ICT relies on electricity, and thus, its development may also decrease the TFCEE. With the operation of technologies, e.g., 5G, cloud computing, and data centers, there is a need for a growing number of energy-intensive infrastructures (17). Furthermore, applying ICT in various fields may create more demand for ICT-related products and services, exerting pressure on energy demand and increasing carbon emissions (24).

Notwithstanding, ICT advancement directly improves the TFCEE in two ways. First, ICT promotes the low-carbon transition of people's work and lifestyles. For instance, paperless transmission, e-commerce, remote offices, and the sharing economy are gaining ground along with the development of ICT. These approaches achieve more effective utilization of resources and reduce emissions of CO_2_ caused by logistics, transportation, and the functioning of building space (36), thereby directly improving the TFCEE. Second, ICT improvement substantially boosts the level of informatization in other industries. Regarding production, ICT plays a fundamental role in the practical implementation of an energy management system (EMS) (37). The EMS, in turn, automatically controls, adapts, and optimizes the energy networks and orderly configures and schedules the multiple energy systems, thereby increasing the efficiency of the energy system.

Summing up, most studies suggest a generally beneficial influence of ICT development on the TFCEE. In other words, extant empirical evidence indicates that the positive impact of ICT tends to outweigh its adverse side effects on the TFCEE (24). Accordingly, we propose hypothesis 1, stated below.

**Hypothesis 1**. ICT development positively affects the TFCEE.

### 3.2 The mediation effect of green innovation

ICT development is conducive to green innovation via the generation, acquisition, processing, and exchange of information (38). Specifically, ICT expansion drives the growth of information related to environmentally sound technologies while improving search efficiency and decreasing the acquisition cost. Moreover, ICT advances dramatically enhance the ability of individuals and organizations to process information and transform enterprises from experience- to data-driven (39). ICT also forms innovation clusters by linking various innovative entities (e.g., firms, universities, R&D institutions, and non-governmental organizations) together, thus facilitating communication, complementarity, and knowledge spillover among innovators (38). The mechanisms mentioned above increase the utilization of relevant information resources and the success rate of green innovation, thus promoting green innovation.

Green innovation, in turn, can significantly improve the TFCEE through the following mechanisms. First, some green technological advances (e.g., capturing and storing CO_2_) directly contribute to energy savings and carbon reduction (10). Likewise, air quality management and energy storage technologies enable the same output with less energy input and fewer emissions, leading to a higher TFCEE. Second, environmental innovations can help renovate and upgrade pollution treatment equipment (40). As such, assuming it is effectively adopted, green technology innovation optimizes production processes and promotes the low-carbon transformation of energy-intensive enterprises, thereby improving the TFCEE (41). Given the above backdrop, we propose a second hypothesis as follows.

**Hypothesis 2**. ICT development significantly increases the TFCEE by promoting green innovation.

### 3.3 The mediation effect of energy structure optimization

ICT has motivated the so-called energy internet development (42), which uses cyberspace to identify, collect, analyse, transmit, and manage energy information. Furthermore, the energy internet integrates internet technology, multi-energy networks and technologies for renewable energy generation, enhancing system resilience and energy utilization efficiency and promoting renewable energy penetration (43). Additionally, the energy internet can overcome barriers to extraction, transmission, and control of large-scale clean energy projects and promote the transformation of economic development from traditional fossil fuels to a clean and efficient energy source (42). This process, in turn, drives the sustainable utilization of energy resources and promotes energy structure optimization. Moreover, with the growing popularity of the energy internet, clean energy usage is continuously expanding (e.g., electric vehicles), which further optimizes the energy structure.

Summing up, energy structure optimization increases the proportion of clean energy sources. Therefore, energy structure optimization can achieve the same economic output with fewer carbon emissions, improving the TFCEE (44). Given the above, we put forth hypothesis 3.

**Hypothesis 3**. ICT development significantly increases the TFCEE by promoting energy structure optimization.

## 4 Dataset, variables, and models

### 4.1 Benchmark regression model

The relationship between ICT development and the TFCEE can be represented by the basic model as follows.


(1) $$\begin{array}{l} TFCEE_{i,t}=\alpha_{0}+\alpha_{1}ICT_{i,t}+\beta \;Control_{i,t}+\mu_{i}+\delta_{t}+\varepsilon_{i,t} \end{array}$$

Where *TFCEE*_*i, t*_ is the total-factor carbon emission efficiency in Chinese province *i* and year *t*. The *ICT* term represents ICT development, while the *Control* term represents a vector of five control variables, including provincial population (*POP*), science and technology expenditure intensity (*TECH*), economic development (*GDP*), degree of economic openness (*OPEN*) and industrial structure (*IND*). μ_*i*_ and δ_*t*_ are the province and time-fixed effects, respectively. ε_*i, t*_ is a random error term with zero mean, constant variance and is not serially correlated.

Given that the outcome variable *TFCEE* may be path-dependent (autoregressive process) (12, 29), we introduce a first lag of the dependent variable (*TFCEE*_*i, t*−1_) into the right-hand side of Equation (1). However, dynamic models tend to suffer from endogeneity issues. In contrast, the OLS and the Fixed Effects (FE) estimators would yield biased results (45). Therefore, we use 2SGMM^2^ (46) to estimate the dynamic model [see Equation (2)], which tests hypothesis 1.


(2) $$\begin{array}{l} TFCEE_{i,t}=\alpha_{0}+\alpha_{1}TFCEE_{i,t-1}+\alpha_{2}ICT_{i,t}+\beta \;Control_{i,t}\\ \;+\mu_{i}+\delta_{t}+\varepsilon_{i,t} \end{array}$$

### 4.2 Mediation effects

Besides its direct effect, ICT development may also have two indirect effects on TFCEE, generally referred to as the mediation effects. To empirically examine such effects, Equation (3) is constructed as follows (47) to test hypotheses 2 and 3.


(3) $$\begin{array}{l} MED_{i,t}=\lambda_{0}+\lambda_{1}MED_{i,t-1}+\lambda_{2}ICT_{i,t}+\theta \;Control_{i,t}\\ \;+\mu_{i}+\delta_{t}+\varepsilon_{i,t} \end{array}$$

Where *MED* represents two mediator variables: green innovation (*GINNO*) and energy structure (*ENER*). If the coefficient of λ_1_is significant, a mediation effect exists, and vice versa (47).

### 4.3 Dynamic threshold model

With changes in green innovation (*GINNO*) and energy structure (*ENER*), the impact mechanism between ICT development and the TFCEE may have a structural change—that is, there may be a threshold effect (30). To examine this issue, we construct a dynamic threshold model (30) and present it below in Equation (4).


(4) $$\begin{array}{l} TFCEE_{i,t}=\alpha_{0}+\alpha_{1}TFCEE_{i,t-1}+\alpha_{2}ICT_{i,t}\left(q_{i,t}\leq T\right)\\ \;+\;\alpha_{3}ICT_{i,t}\left(q_{i,t}>T\right)+\beta \;Control_{i,t}+\mu_{i}+\delta_{t}+\varepsilon_{i,t} \end{array}$$

Where *q*_*i, t*_ is the threshold variable, including green innovation (*GINNO*) and energy structure (*ENER*). *T* represents the threshold value to be estimated. α_2_(α_3_) represents the effect of ICT development on the TFCEE when the threshold variable is below (above) the threshold value.

### 4.4 Data and variables

In line with prior studies (23, 48), we use the NDDF to estimate the *TFCEE* variable. We use a sample of 30 Chinese provinces (*n* = 1, 2, …, 30). Each province includes inputs of capital stock (*K*), calculated by the perpetual inventory method; labor (*L*), measured by the total number of employees; and energy (*E*), expressed as the total energy consumption. Desired output GDP (*Y*) is proxied by provincial real GDP. In contrast, the undesired output of carbon emissions (*C*) is calculated according to the 2006 Intergovernmental Panel on Climate Change guidelines for national greenhouse gas inventories. Assuming that the optimal solution of each input and output obtained through the NDDF linear programming is $\beta_{K}^{*},\beta_{L}^{*},\beta_{E}^{*},\beta_{Y}^{*},\beta_{C}^{*}$. The dependent variable *TFCEE* can be formulated in Equation (6) (48).


(5) $$\begin{array}{l} TFCEE=\frac{\frac{1}{4}\left[\left(1-\beta_{K}^{*}\right)+\left(1-\beta_{L}^{*}\right)+\left(1-\beta_{E}^{*}\right)+\left(1-\beta_{C}^{*}\right)\right]}{1+\beta_{Y}^{*}}\\ \;=\frac{1-\frac{1}{4}\left[\beta_{K}^{*}+\beta_{L}^{*}+\beta_{E}^{*}+\beta_{C}^{*}\right]}{1+\beta_{Y}^{*}} \end{array}$$

*TFCEE* ranges from 0 to 1, such that a higher value of the *TFCEE* indicates higher production efficiency of the province. Consequently, if provincial *TFCEE* were equal to 1 (0), it would mean that the corresponding province was fully efficient (inefficient).

We construct a comprehensive index to measure ICT advancement, in line with recent studies (49), as shown in Table 1. Additionally, we employ the entropy method to evaluate the ICT development index (*ICT* variable), which ranges from 0 to 1.

**Table 1:** ICT development evaluation.

**Variable**	**Dimension**	**Indicators**	**Unit**
ICT development index	ICT basic resources	Netizen penetration rate	%
		Mobile phone ownership per 100 people	—
		IPv4 proportion^a^	%
	ICT information resources	Number of domain names per 10,000 people	—
		The average size of a webpage in kilobytes	KB
		The average number of websites per company	—
	ICT applications	The proportion of employment in ICT-related industries	%
		The ratio of e-commerce transactions to GDP	%
		Express service delivery (after placing an order online) volume per person	—

^a^IPv4 is the fourth version of the Internet Protocol (IP), and its proportion can reflect the ICT infrastructure construction level (43).

Descriptions of all the variables are presented in Table 2. All variables are at the provincial level. The selection and measurement of the variables align with the prior research (6, 11, 16, 50, 51).

**Table 2:** Variable descriptions.

**Variable type**	**Variable**	**Symbol**	**Variable measurement**
Dependent variable	Total-factor carbon emission efficiency	*TFCEE*	As described in Section 4.4
Explanatory variable	Information and communication technology development	*ICT*	As described in Section 4.4
Control variable	Economic development	*GDP*	Natural logarithm of the per capita GDP
	Population	*POP*	Natural logarithm of the population
	Economic openness	*OPEN*	The ratio of the total value of imports and exports to GDP
	Industrial structure	*IND*	The ratio of secondary industries added value to GDP
	Urbanization level	*URBAN*	Urban population as a percentage of the total population
	Government financial autonomy	*GOVAU*	The ratio of government general budget expenditure to GDP
Mediator variable	Green innovation	*GINNO*	Number of green patent applications per capita
	Energy structure	*ENER*	Share of coal in total energy consumption
Public health measures	Inpatients	*INP*	The ratio of inpatients to the total population
	Mortality	*MOR*	Resident mortality (per 1,000 persons)
	Maternal Mortality	*MMOR*	Maternal mortality (per 1,000 live births)

The sample ranges from 2008 to 2019. Due to limited data availability in Hong Kong, Macao, Taiwan, and Tibet, this study focuses on the other 30 provincial administrative regions as the sample. We obtained data from the National Bureau of Statistics, the China Statistical Yearbook, the Statistical Report on China's internet development, and the Chinese Research Data Services Platform. All the data are adjusted for inflation with 2008 as a base period to ensure comparability.

Table 3 presents descriptive statistics for variables. We can observe that the dependent variable *TFCEE* has the minimum (maximum) value of 0.177 (1), with a mean and standard deviation of 0.430 and 0.157, respectively. The leading explanatory variable, *ICT*, has a minimum (maximum) value of 0.018 (0.773), with a mean and standard deviation of 0.116 and 0.117. This result suggests substantial cross-provincial variability in the *TFCEE* and *ICT* during the sample period.

**Table 3:** Descriptive statistics.

**Variable type**	**Variable**	**Observations**	**Mean**	**Std. Dev**.	**Min**	**Max**
Dependent variable	*TFCEE*	360	0.4399	0.1571	0.1766	1.000
Explanatory variable	*ICT*	360	0.1162	0.1167	0.0183	0.7728
Control variable	*GDP*	360	10.5173	0.5008	9.0812	11.7410
	*POP*	360	8.1924	0.7395	6.3172	9.3519
	*OPEN*	360	0.2711	0.2967	0.0114	1.5914
	*IND*	360	0.4507	0.0864	0.1620	0.6150
	*URBAN*	360	0.5639	0.1319	0.2911	0.8960
	*GOVAU*	360	0.2758	0.1266	0.0870	0.7724
Mediator variable	*GINNO*	360	1.3900	2.1483	0.0548	16.0956
	*ENER*	360	0.4145	0.1520	0.0121	0.7241
Public health measures	*INP*	360	0.1058	0.0340	0.0391	0.1922
	*MOR*	360	6.0423	0.7632	4.2100	7.5700
	*MMOR*	360	0.1624	0.1004	0.0110	0.6204

**Figure 2:**
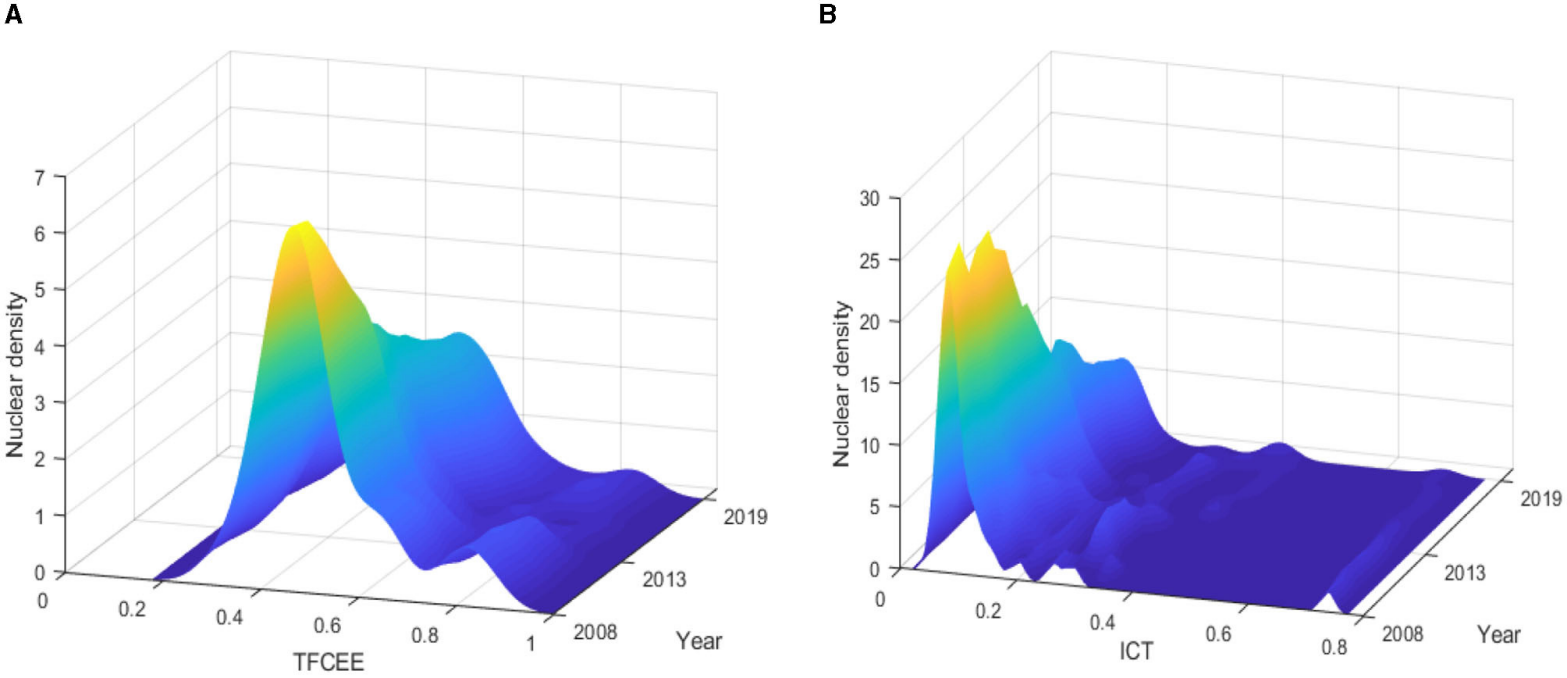
Trends of TFCEE **(A)** and ICT **(B)** in China from 2008 to 2019. Source: Authors' calculation.

Figure 2 illustrates the trends of *TFCEE* (Figure 2A) and *ICT* (Figure 2B) in China from 2008 to 2019. We can observe that *TFCEE* and *ICT* follow a gradually increasing path over time.

## 5 Results and discussions

### 5.1 Benchmark regression results

Table 4 shows the estimated results of the dynamic panel regressions corresponding to the benchmark model in Equation (2). Columns (1) and (2) display the estimation results using the OLS and the FE methods. Column (3) shows the output from the 2SGMM estimation technique. The coefficient on the *LTFCEE* in column (3) is 0.9755 and larger (smaller) than the estimated coefficient from the FE (OLS) method. The results based on the 2SGMM model also pass the m1, m2, and Hansen tests. Due to the above, we can assume that our 2SGMM model is correctly specified. Furthermore, the coefficient on the *LTFCEE* term in column (3) is positive (within the range of zero to one) and statistically significant. This finding indicates that Chinese provincial-level *TFCEE* is path-dependent, in line with the recent study (12, 29). Such evidence could mean that because of the difficulty of changing the provincial-level production mode in the short term, the TFCEE changes slowly and exhibits path dependence.

**Table 4:** Benchmark regression results.

**Method of estimation**	**OLS**	**FE**	**2SGMM**
**Variables**	**(1)**	**(2)**	**(3)**
*TFCEE*	0.9926^***^	0.9075^***^	0.9755^***^
	(0.0161)	(0.0280)	(0.0107)
*ICT*	0.0299^*^	0.0794^*^	0.0404^***^
	(0.0162)	(0.0443)	(0.0097)
*GDP*	−0.0037	−0.0094	−0.0023
	(0.0062)	(0.0313)	(0.0066)
*POP*	0.0041	−0.0302	0.0054^***^
	(0.0026)	(0.0218)	(0.0018)
*OPEN*	0.0107^*^	0.0729	0.0121^***^
	(0.0056)	(0.0714)	(0.0032)
*IND*	−0.0109	0.0227	−0.0174
	(0.0143)	(0.0320)	(0.0111)
*URBAN*	0.0292	−0.1574	0.0281
	(0.0223)	(0.1542)	(0.0217)
*GOVAU*	−0.0137	−0.0653^**^	−0.0141
	(0.0158)	(0.0258)	(0.0114)
Adj R^2^	0.9918	0.9695	
m1 (Z-statistic)			−1.66^*^
m2 (Z-statistic)			0.89
Hansen test (*p*-value)			0.18
N	330	330	330

The dependent variable is the TFCEE. ^*^, ^**^, and ^***^ are the 10%, 5%, and 1% statistical significance levels of the estimated coefficients, respectively.

Column (3) also shows that the *ICT* variable coefficient is statistically significant and positive. Specifically, the coefficient informs us that, on average, for every 10 per cent increase in the ICT development index, we expect the *TFCEE* to increase by 0.11 per cent, ceteris paribus.^3^ Therefore, Table 4 implies that ICT development significantly promotes TFCEE improvement, which supports hypothesis 1. This finding supports the conclusions of Hao et al. (30) and Wu et al. (31) about the role of ICT development in driving low-carbon development. The estimated results can be associated with ICT accelerating the intelligent and exemplary management of the whole production process, improving the efficiency of resource allocation, reducing inefficient energy consumption and carbon emissions, and thus improving the TFCEE. China has entered a phase where the pace of economic growth has slowed down. In contrast, the task of carbon emission reduction is urgent due to the associated environmental and health damage, as well as policy commitments, thereby causing the TFCEE improvement to encounter bottlenecks. As such, our findings could inspire strategic planning development in China and across other emerging economies with similar goals and problems.

### 5.2 The mediation effects of green innovation and energy structure

Environmental innovations promote continuous production process improvement, e.g., by avoiding high carbon emissions at source and fostering the development of end-of-pipe treatment technologies (carbon-storing and reusing) (41). Therefore, green innovation significantly reduces energy saving and carbon reduction costs, thus driving the TFCEE improvement.

Column (1) in Table 5 shows the estimated results from 2SGMM regression models testing for indirect ICT effects in the TFCEE using green innovation as a mediator variable from equation (3). The coefficient on the *ICT* variable is significant and carries a positive sign, meaning that ICT progress increases green innovation. This aligns with Tang et al. (38), who adopt China's broadband policy as a quasi-natural experiment. Columns (2) and (3) further distinguish between high-quality innovation represented by green inventions (*GINV*) and low-quality innovation represented by green utility models (*GUTI*), respectively. Estimated coefficients indicate that ICT advancement has a significantly stronger (statistically and economically) effect on promoting high-quality rather than low-quality green innovation. Such results can be associated with the ICT promoting digital ecology, thereby increasing research and development (38). This comparative advantage, in turn, makes it more likely for enterprises to receive long-term external financial support when conducting quality green innovation research characterized by higher difficulty levels, greater failure rates, and more extended periods (53). Such incentives motivate Chinese enterprises and significantly increase high-quality green innovation.

**Table 5:** Mediation effect verification results.

**Variables**	***GINNO***	***GINV***	***GUTI***	***ENER***
	**(1)**	**(2)**	**(3)**	**(4)**
*Lagged dep. Var*.	0.9427^***^	0.9790^***^	0.9889^***^	0.8687^***^
	(0.0321)	(0.0172)	(0.0839)	(0.0711)
*ICT*	2.8247^***^	1.7897^***^	0.7415^*^	−0.0778^**^
	(0.6099)	(0.4265)	(0.4127)	(0.0316)
Controls	Yes	Yes	Yes	Yes
m1 (Z-statistic)	−2.39^**^	−2.26^**^	−1.83^*^	−2.98^***^
m2 (Z-statistic)	−0.42	−0.69	0.03	0.64
Hansen test	0.85	0.94	0.80	0.29
N	330	330	330	330

The dependent variables in columns (1) to (4) are overall green innovation, green inventions, green utility models, and energy structure, respectively. ^*^, ^**^, and ^***^ are the 10%, 5%, and 1% statistical significance levels of the estimated coefficients, respectively. We use the same set of control variables as in Equation (2). However, for this study's brevity, these coefficients are not presented but are available upon request.

Because ICT affects green innovation, which, in turn, affects the TFCEE, we can assume that ICT development indirectly affects the TFCEE, which supports hypothesis 2. ICT is deeply integrated into enterprises' production and operation activities, promoting their forward-looking technology research and significantly enhancing green innovation capabilities (54). At the same time, ICT development can enable firms to obtain knowledge and information at a lower marginal cost, break through barriers between industries, and further promote green innovation via the knowledge spillover effects (38). Green innovation, in turn, effectively increases energy efficiency and improves production processes (41), enhancing the TFCEE.

The optimization of the energy structure implies an increase (decrease) in the proportion of renewable energy (fossil fuels) in energy consumption. This can reduce carbon emissions, driving the TFCEE's improvement. The testing results for indirect ICT effects in the TFCEE using energy structure as a mediator variable from equation (3) are displayed in column (4) in Table 5. We can observe that the estimated coefficient on the *ICT* variable is negative and significant, which means that ICT development reduces coal's share in total energy consumption, i.e., optimizes the energy structure. This corroborates Xu et al. (55), who found that improving digitalization improves energy composition in a global study of 109 countries.

Again, because the ICT affects energy structure, which, in turn, promotes an improvement in the TFCEE, we can assume that the former indirectly affects TFCEE, in line with hypothesis 3. Specifically, ICT changes energy production and consumption patterns through knowledgeable information-based systems while accelerating energy transformation (27). Additionally, the ICT provides technological support for renewable energy development (43), promoting global *energy internet* construction (42). This helps reduce the use of traditional high-carbon energy sources, fosters the growth of clean energy sources and optimizes the energy structure. Increasing the proportion of clean and renewable energy helps cut carbon emissions, improving the TFCEE.

### 5.3 Dynamic threshold model results

In this section, we test whether the threshold effect exists in the ICT-TFCEE nexus. The threshold variables include green innovation (*GINNO*) and energy structure (*ENER*). In the first step, we conduct 1,000 repetitions of bootstrap self-sampling. Table 6 displays the self-sampling test results of the threshold value and a corresponding confidence interval. The Wald statistics and *p*-values all pass the significance test, informing that our model is suitable for further analysis.

**Table 6:** Test of the threshold effect.

**Threshold variables**	**Dynamic threshold model**	**Threshold value**	**Wald statistics**	***p*-Value**	**BS**	**95% confidence interval**
*GINNO*	2SGMM	0.8480	607.8981	0.000	1,000	(0.0910, 6.0144)
*ENER*	2SGMM	0.4769	542.3234	0.000	1,000	(0.1242, 0.6421)

Having estimated the threshold values for *GINNO* and *ENER* variables in Table 6, we run 2SGMM dynamic threshold panel regressions corresponding to Equation (4). The estimated results appear in Table 7, where *q*_*it*_ ≤ C (*q*_*it*_ > C) indicates that the threshold variable is below or equal to (above) the threshold value. Column (1) shows the results with green innovation (*GINNO*) as the threshold variable. We can observe that when green innovation exceeds the threshold value, the estimated coefficient on *ICT* changes from 0.1064 to 0.1737 and remains statistically significant. For instance, at *q*_*it*_ ≤ C (*q*_*it*_ > C), for every 10 per cent increase in *ICT*, we would expect the TFCEE to increase on average by 0.29 (0.47) per cent, holding all else constant. This result can be associated with the evidence that at the early stage of green innovation development, enterprises' research costs are high, and green patent application in production is still being explored. Because of that, the potential for TFCEE improvement through ICT development is somewhat limited. However, once the green innovation development exceeds a certain threshold, key technical challenges to improve the TFCEE no longer hold (38). This, in turn, decreases carbon emissions reduction costs and provides new impetus for ICT development to promote the TFCEE.

**Table 7:** Dynamic threshold panel regression results.

**Variables**	***GINNO***	***ENER***
	**(1)**	**(2)**
*Lagged dep. Var*.	0.8351^***^	0.8864^***^
	(0.0449)	(0.0143)
*ICT(q_*it*_ ≤ C)*	0.1064^*^	0.1434^***^
	(0.0577)	(0.0168)
*ICT(q_*it*_>C)*	0.1737^***^	0.0932^**^
	(0.0552)	(0.0370)
Controls	Yes	Yes
m1 (Z-statistic)	−1.67^*^	−1.65^*^
m2 (Z-statistic)	0.82	0.84
Hansen test	0.70	0.99
N	330	330

The dependent variable is the TFCEE. ^*^, ^**^, and ^***^ are the 10%, 5%, and 1% statistical significance levels of the estimated coefficients, respectively. We use the same set of control variables as in Equation (2). However, for this study's brevity, these coefficients are not presented but are available upon request.

Column (2) of Table 7 displays estimated results with the energy structure (*ENER*) as the threshold term. Since *ENER* is represented by the share of coal in total energy consumption, a decrease in *ENER* implies optimization of the energy structure. We can observe that as *ENER* changes from above to below its threshold value, the coefficient on the *ICT* increases from 0.0932 to 0.1434 and remains statistically significant. The fossil fuel-based energy structure is associated with substantial carbon emissions, which impedes the TFCEE improvement. For instance, some underdeveloped regions in northwest China are highly dependent on fossil fuels, which will weaken their capacity to improve the TFCEE (56). As the cost of renewable energy decreases and the proportion of fossil fuels falls below a certain threshold, a cleaner energy structure can strengthen the relationship between ICT and TFCEE (44).

### 5.4 The health co-benefits of the TFCEE improvement

Excessive inhalation of carbon dioxide harms the respiratory system and reduces higher-level cognitive abilities (7), directly leading to adverse health effects. CO_2_ can also indirectly have negative effects on health. For example, carbon dioxide decreases the content of essential nutrients for humans in crops (57); global warming exacerbates air pollution, increases the frequency of extreme weather events, and damages ecosystems (6). Increasing the TFCEE leads to lower carbon emissions for the same output, potentially decreasing adverse health effects on Chinese residents. However, whether the improvement in TFCEE directly influences public health in China has not been empirically tested.

Columns (1–3) of Table 8 display the output from 2SGMM regression models testing for the impact of TFCEE on the three indicators of public health: the ratio of inpatients to total population (*INP*), the mortality rate per 1,000 persons (*MOR*), and maternal mortality rate per 1,000 births (*MMOR*).^4^ Higher values of these indicators indicate lower levels of public health and vice versa. We can observe that the coefficients of the *TFCEE* variable in all columns are significantly negative, indicating that TFCEE improves health levels. Accordingly, our analysis offers nascent robust empirical evidence supporting the health co-benefits of ICT driving TFCEE improvement.

**Table 8:** Health co-benefits examination.

**Variables**	***INP***	***MOR***	***MMOR***
	**(1)**	**(2)**	**(3)**
*Lagged dep. Var*.	0.8956^***^	0.7685^***^	0.8617^***^
	(0.0866)	(0.1053)	(0.1880)
*TFCEE*	−0.0108^*^	−2.4048^**^	−0.2343^**^
	(0.0058)	(1.0470)	(0.1111)
Controls	YES	YES	YES
m1 (Z-statistic)	−2.26^**^	−2.66^***^	−1.80^*^
m2 (Z-statistic)	1.32	0.53	−1.56
Hansen test	0.34	0.65	0.76
N	330	330	330

The dependent variables in columns (1) to (3) are inpatients, mortality, and maternal mortality rate, respectively. ^*^, ^**^, and ^***^ are the 10%, 5%, and 1% statistical significance levels of the estimated coefficients, respectively.

### 5.5 Robustness test

To confirm the reliability of the estimated results presented in Table 4 and the associated research findings, we test their robustness concerning the following four aspects. First, we recalculate the TFCEE using the slack-based measure model (58). Second, we replace the core explanatory variable (*ICT*). Specifically, we adopt the principal component analysis (PCA) to recalculate the new ICT development index (*ICT_PCA*). Third, this study re-estimates the benchmark model using the difference GMM estimator. Finally, we rerun the benchmark model after shortening the sample period by 1 year, from 2008–2019 to 2009–2019, to account for the potential impact of the 2008 great financial crisis.

Results corresponding to the four robustness procedures are shown in Table 9. The coefficients on the *TFCEE* remain statistically significant, carry positive signs, and range from 0.6740 to 0.9512 in columns (1) and (2), respectively. Furthermore, the estimated coefficients on the ICT development variable in all four columns remain statistically significant and positive. Overall, Table 9 confirms that the results and research conclusions from Section 5.1 are robust and further corroborate hypothesis 1.

**Table 9:** Robustness tests.

**Variables**	**Alternative dependent variable**	**Alternative explanatory variable**	**Difference GMM**	**Shorter period of analysis**
	**(1)**	**(2)**	**(3)**	**(4)**
*LTFCEE*	0.6740^***^	0.9512^***^	0.7936^***^	0.9329^***^
	(0.0483)	(0.0127)	(0.0257)	(0.0286)
*ICT*	0.2797^***^		0.0513^*^	0.0780^***^
	(0.0887)		(0.0255)	(0.0235)
*ICT_PCA*		0.0063^***^		
		(0.0012)		
Controls	Yes	Yes	Yes	Yes
m1 (Z-statistic)	−2.43^**^	−1.66^*^	−1.84^*^	−1.66^*^
m2 (Z-statistic)	−0.20	0.89	0.96	0.84
Hansen test	0.42	0.18	0.87	0.24
N	330	330	300	300

The dependent variable is the TFCEE. ^*^, ^**^, and ^***^ are the 10%, 5%, and 1% statistical significance levels of the estimated coefficients, respectively.

## 6 Conclusions and policy implications

Considering the ICT's rapid advancements and the urgency of meeting goals for carbon peaking and net zero, the study takes data from 30 Chinese provinces during the 2008–2019 period. We use the 2SGMM dynamic models to empirically test the impact of ICT development on the TFCEE and the mediation, threshold, and moderating effects, as well as the health co-benefits. This study delivers several significant findings. First, ICT development significantly increases the TFCEE. Second, ICT can indirectly improve TFCEE through green innovation and energy structure optimization. Moreover, when green innovation (energy structure) switches from below to above (above to below) its threshold value, the promotional effect of ICT development on the TFCEE increases substantially in magnitude. Third, TFCEE improvement has significant public health-related co-benefits by significantly reducing the inpatient and mortality ratios.

Three policy implications can be derived from our findings. First, from the perspective of TFCEE improvement, the results suggest policies promoting the cross-industry application of ICT. Primarily, policymakers should focus on the digital and intelligent transformation of energy- and environment-related fields to realize the normalization of ICT applications, decoupling economic growth from carbon emissions and creating new impetus for the promotion of TFCEE. Second, concerning the mediating effects of ICT development on the TFCEE, Chinese decision-makers should foster favorable conditions for realizing the positive ICT effect on green innovation. For instance, they could consider upgrading the intellectual property protection system for green innovation. Moreover, the development of the energy internet should be regarded as an essential direction for energy structure optimization. It is also vital to promote intelligent upgrading of the energy system. Third, from the public health co-benefits perspective, the significant role of TFCEE in decreasing the inpatient and mortality ratios should be emphasized to enhance the motivation of relevant stakeholders in the field of public health toward improving the TFCEE.

Our examination of the ICT-TFCEE nexus is at the provincial level due to the availability of ICT development data. With continuous data enrichment, future research could be conducted at different spatial units, e.g., prefectural and enterprise levels. Moreover, once more data is available, it would be interesting to see whether the recent exogeneous macroeconomic shock of the COVID-19 pandemic and related policies (e.g., lockdowns) affect the documented results and findings. Finally, we advocate further research on the long-run convergence-divergence path in public health across the cities and provinces with different levels of ICT and the TFCEE by applying the distribution dynamics approach (59–61).

## Data availability statement

The original contributions presented in the study are included in the article/supplementary material, further inquiries can be directed to the corresponding author.

## Author contributions

JL: Conceptualization, Data curation, Software, Visualization, Methodology, Writing — original draft, Writing — review & editing. MW: Conceptualization, Data curation, Supervision, Methodology, Writing — original draft, Writing — review & editing, Project administration. TC: Conceptualization, Supervision, Validation, Writing — review & editing, Project administration.

## Footnotes

^1^Hao et al. (30) and Wu et al. (31) examine the threshold effects of ICT/internet development on electricity intensity or green total factor energy efficiency in China.

^2^2SGMM is a highly efficient estimation method in tackling the issue of endogeneity (1).

^3^Because the sample's mean values (see Table 3) of *TFCEE* (0.430) and *ICT* (0.116) and the coefficient on *ICT* (0.0404) in column (3) of Table 4, the average effect on the *TFCCE* associated with a 10 per cent increase in *ICT* variable is equal to 0.116^*^0.1^*^0.0404/0.430, or 0.11 per cent (52).

^4^All three variables are at the provincial level and obtained from the China Health Statistical Yearbook.
